# Carbon price prediction based on a scaled PCA approach

**DOI:** 10.1371/journal.pone.0296105

**Published:** 2024-01-02

**Authors:** Xiaolu Wei, Hongbing Ouyang

**Affiliations:** 1 Business School, Hubei University, Wuhan, 430062, China; 2 Department of Economics, Huazhong University of Science and Technology, Wuhan, 430074, China; Sunway University, MALAYSIA

## Abstract

Carbon price prediction is of great importance to regulators and participants in the carbon trading market. It is the basis for developing policies related to the carbon trading market and stabilizing that market. Considering the numerous factors that influence carbon prices in China, dimensionality reduction is needed to improve the prediction accuracy and efficiency. However, the traditional dimensionality reduction methods fail to fully consider the role of influencing factors, which has certain limitations. In this paper, a new dimensionality reduction method, namely scaled principal component analysis (s-PCA), is employed to improve the prediction accuracy of carbon prices. Firstly, a factor library that influence carbon prices is constructed from three perspectives: technical indicators, financial indicators and commodities indicators. Then, the s-PCA method is used to reduce the dimensionality of factors influencing carbon price. Next, two different methods are used to predict carbon prices, including traditional regression method and Long Short-Term Memory (LSTM) method. Finally, the economic value of the s-PCA method is examined by constructing investment portfolios. The empirical results of the Hubei Emissions Exchange show that the s-PCA model outperforms other competing models both in- and out-of-sample. In addition, the LSTM model could improve the performance of the s-PCA model in carbon price prediction. From a market timing perspective, investors can achieve a greater return and a larger Sharpe ratio using the s-PCA method than using other comparative methods and buy-and-hold strategy. Therefore, the s-PCA method is effective and robust in predicting carbon price.

## Introduction

Global warming and glacial melting caused by carbon emissions are becoming prominent problems, which seriously threaten human food supply and living environment. China, as the largest carbon emitter, plays an essential role in global climate change and is under increasing pressure to control carbon emissions due to its rapid economic growth. Therefore, China comprehensively promotes energy transformation and accelerates the construction of a clean and low-carbon modern energy system. Since 2011, China has established seven major carbon emission exchanges, including the Guangzhou Carbon Emissions Exchange, Shenzhen Emissions Exchange, Beijing Environmental Exchange, Shanghai Environmental Energy Exchange, Hubei Carbon Emissions Exchange, Tianjin Emissions Exchange and Chongqing Carbon Emissions Exchange, which are considered to be important measures to regulate the allocation of carbon emissions and ease the pressure on carbon emissions. With the development of carbon emission trading markets, carbon emission trading in China has gradually become active. Carbon price prediction is increasingly important to understand the development of China’s carbon market and to make decisions about carbon reduction. Therefore, it is essential to choose the appropriate method to improve the accuracy of predicting carbon price.

As the carbon trading market has matured, the research on carbon price prediction has increased sharply at home and abroad. The models for predicting carbon price mainly include generalized autoregressive conditional heteroscedasticity (GARCH) model [1], least square support vector machine (LSSVM) model [2], long short-term memory (LSTM) model [3], empirical mode decomposition (EMD) model [4], extreme learning machine (ELM) model [5] and ensemble learning methods [6,7]. However, studies on carbon price prediction still have some shortcomings: (a) Most of the existing studies predict carbon prices based on technical indicators and ignore the influence of other factors, which has certain limitations. Therefore, this paper predicts carbon prices by constructing a factor library containing technical indicators, financial indicators and commodities indicators. (b) Due to the large number of factors affecting carbon price, dimensionality reduction methods are very useful which could reduce these factors to a few combinations. However, one recognized weakness of traditional dimensionality reduction methods, such as principal component analysis (PCA), is that it ignores the target information target completely. In this paper, we employ the scaled principal component analysis (s-PCA) approach proposed by Huang et al. [8] to predict carbon prices. The s-PCA approach is a variant of the PCA approach by further incorporating supervised learning. Specifically, before extracting diffusion indexes, the s-PCA approach uses the regression coefficient of the prediction target on each predictor to scale the corresponding predictor. Therefore, the s-PCA approach has the potential to improve the predictability by considering the target information in the process of dimensionality reduction. (c) Most of the existing studies have explored the effectiveness of the dimensionality reduction methods through linear regression models. However, the results of these studies are not robust due to the non-linear and non-stationary nature of the carbon price series. In this paper, we will use both linear regression model and LSTM model to further explore the effectiveness of the s-PCA approach in carbon price prediction. (d) Most of the existing studies focus on the European carbon allowance (EUA), while rarely analyzing China’s carbon trading market. Given this, this paper will conduct an empirical study on carbon price prediction in China.

In this paper, we investigate the carbon price predictability of the s-PCA model based on a factor library containing technical indicators, financial indicators and commodities indicators. The carbon price data of Hubei Carbon Emissions Exchange is used as the research object for empirical study. To demonstrate the superiority of the s-PCA model, it is compared with the PCA model and PLS model. The main contributions and innovations of this paper are listed below:

- The s-PCA model is applied to predict carbon price. The diffusion indexes obtained from s-PCA is adopted to predict carbon price, which improves the computational efficiency and prediction accuracy.
- We construct a factor library of carbon prices, including technical indicators, financial indicators and commodities indicators, to further improve the interpretability of carbon price prediction.
- Combining the s-PCA model with linear regression method and LSTM model, two hybrid models of carbon price prediction are proposed in this paper, which might provide a new idea to test the effectiveness of the dimensionality reduction method, and effectively make up for the shortcomings of carbon price prediction based on linear regression models.
- Hubei Carbon Emissions Exchange is used as the research sample for empirical analysis, while two other related models are used for performance comparison to prove the effectiveness of the s-PCA model. In addition, the evaluation metrics used in this paper include R2, RMSE, MAE, and DM.

The remainder of this paper is structured as follows: Section 2 describes the methods used in this paper. Section 3 provides the empirical results and discussion based on the carbon price data of the Hubei Emission Exchange. Section 4 presents a series of robustness tests to verify the prediction performance of the s-PCA model. Section 5 describes the results of market timing, which confirm the economic value of the s-PCA model employed in this paper. Finally, Section 6 provides the conclusions and future work.

## Data and methodology

### Data

#### Carbon prices

**Fig 1:**
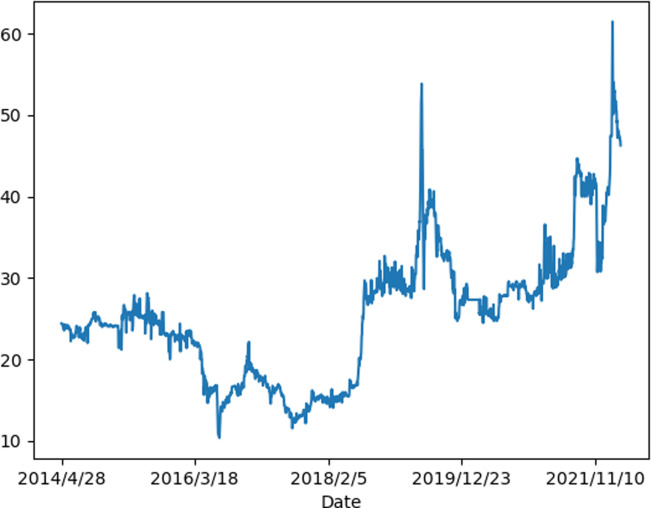
The carbon price of Hubei.

To accelerate the progress of emission peak and carbon neutrality, China has established eight carbon emissions trading pilots, including Shenzhen, Guangdong, Hubei, Tianjin, Shanghai, Chongqing, Beijing and Fujian. Among them, Hubei Carbon Emissions Exchange has the largest trading scale and the highest market participation. The cumulative volume of carbon emission allowances (CEA) traded in the Hubei Carbon Emissions Exchange has reached 360 million tons, with a cumulative turnover of 8.7 billion yuan. In addition, there are a total of 332 emission control enterprises in the Hubei Carbon Emissions Exchange, accounting for more than 70% of the secondary industry’s total value. Due to the value of in-depth research, Hubei Carbon Emissions Exchange is chosen as the research object for empirical analysis in this paper. Table 1 shows the statistical descriptions of the carbon price. Fig 1 depicts the general trend of carbon price in the Hubei Carbon Emissions Exchange. It can be seen that the carbon price of Hubei is highly non-linear and volatile.

**Table 1:** Statistical descriptions of the carbon price in Hubei.

Variable	Mean	Minimum	Maximum	Standard deviation	Skewness	Kurtosis
Carbon price	25.38	10.38	61.48	8.18	0.71	0.72

We collect the daily carbon price data of Hubei Carbon Emissions Exchange from the http://k.tanjiaoyi.com/. The data covers the sample period from April 28, 2014 to March 23, 2022. The data with zero transaction volume is removed from the sample. The cleaned sample is divided into three parts. The first part is the training set (60% of the sample), which is used to train the prediction model. The second part is the validation set (20% of the sample), which is used to tune hyper parameters. The third part is the testing set, which is used to evaluate the performance of the prediction model.

#### Indicators selection

By extensive literature, this paper finds that the factors affecting carbon prices mainly include three categories: technical factors, financial factors, and commodity factors. Therefore, this paper employs 71 technical indicators, 13 financial indicators and 25 commodity indicators to predict carbon price. The data of these indicators are collected from the Wind platform and other official website.

Specifically, the 71 technical indicators are based on five popular technical strategies. The first strategy is the momentum (MOM) rule, which constructs a buy or sell signal by comparing the current carbon price and the price k days ago,

$$S_{t,MOM}=\left\{ \begin{array}{c} 1,\mathrm{if}\;P_{t}\geq P_{t-k}\\ 0,\mathrm{if}\;P_{t}<P_{t-k} \end{array}\right.,$$ (2.1)

where P_t_ denotes the carbon price for day t. Following Wang et al. [9], we analyze MOM technical indicators with k=1, 3, 6, 9, 12.

The second strategy is the filtering (FR) rule, in which a buy or sell signal is given by

$$S_{t,FR}^{buy}=\left\{ \begin{array}{c} 1,if\;P_{t}\geq (1+\frac{\mu }{100})\times min(P_{t-1},P_{t-2},\cdots ,P_{t-k})\\ 0,otherwise \end{array},\right.$$ (2.2)

$$S_{t,FR}^{sell}=\left\{ \begin{array}{c} 1,if\;P_{t}\leq (1+\frac{\mu }{100})\times max(P_{t-1},P_{t-2},\cdots ,P_{t-k})\\ 0,otherwise \end{array},\right.$$ (2.3)

where we use ten FR indicators with μ = 5, 10 and k=1, 3, 6, 9, 12.

The third strategy is the moving average (MA) rule, which compares two moving average value and generate a trading signal at the end of day t,

$$S_{t,MA}=\left\{ \begin{array}{c} 1,if\;{MA}_{s,t}\geq {MA}_{l,t}\\ 0,otherwise \end{array}\right.,$$ (2.4)

where

$${MA}_{j,t}=\left(\frac{1}{j}\right)\sum_{i=0}^{j-1}P_{t-i},j=s,l.$$ (2.5)

In this paper, we use six MA indicators with s = 1, 3, 6 and l = 9, 12.

The forth strategy is the oscillator (OSLT) rule, in which a buy or sell signal is produced by

$$S_{t,OSLT}^{buy}=\left\{ \begin{array}{c} 1,if\;RSI\leq 50+\mu \\ 0,otherwise \end{array}\right.,$$ (2.6)

$$S_{t,OSLT}^{sell}=\left\{ \begin{array}{c} 1,if\;{RSI}_{t}\geq 50+\mu \\ 0,otherwise \end{array},\right.$$ (2.7)

where

$$RSI(k)=\frac{Up}{Up+Down}\times 100,$$ (2.8)

Up denotes the magnitude of the upward stock price movement over k days, Down denotes the magnitude of the downward stock price movement over k days, and Up+Down denotes the total magnitude of the stock price movement over the period. We use ten OSLT indicators with μ = 5, 10 and k = 1, 3, 6, 9, 12.

The fifth strategy is the support resistance (SR) rule, where a trading signal is given by

$$S_{t,SR}^{buy}=\left\{ \begin{array}{c} 1,if\;P_{t}\geq (1+\frac{\mu }{100})\times max(P_{t-1},P_{t-2},\cdots ,P_{t-k})\\ 0,otherwise \end{array}\right.,$$ (2.9)

$$S_{t,SR}^{sell}=\left\{ \begin{array}{c} 1,if\;P_{t}\leq (1+\frac{\mu }{100})\times min(P_{t-1},P_{t-2},\cdots ,P_{t-k})\\ 0,otherwise \end{array}.\right.$$ (2.10)

In this study, we analyze ten SR indicators with μ = 5, 10 and k = 1, 3, 6, 9, 12.

Moreover, the 13 financial indicators and 25 commodity indicators are selected from previous typical literature where these indicators show considerable predictive power in carbon price forecasting. The 13 financial indicators include secondary market interest rates for 3-month Treasury bill, 10-year national bond rate [9], S&P 500 index, Dow Jones Composite Index, Shanghai Composite Index, Shenzhen Composite Index, 5-Year Bond Index Yield, WilderHill New Energy Global Innovation Index (NEX), WilderHill Clean Energy Index (CEI) [10], AAA-rated corporate bond spreads, daily spread of 1-year Treasury bill and 10-year government bond [11], USD/CNY and China’s Economic Policy Uncertainty Index [12].

The 25 commodity indicators include ICE-UK natural gas continuous futures price (UKGP), Asia gas price (JKM), S&P GSCI Gas oil index excess return (GGO) [10], ICE-coal Rotterdam continuous futures price (GP), ICE-Brent crude oil continuous futures price (BOP) [13], S&P GSCI Crude oil index excess return (GCO), EUA price, China Electricity Price index and 17 S&P GSCI non-energy commodity indexes (GGOL, GSIL, GALU, GCOP, GLEA, GNIC, GZIN, GCOC, Gcof, Gcor, GCOT, Gsoy, Gsug, Gwhe, GFC, GLH, GLC) [14].

In addition, it should be emphasized that the collection and analysis methods are complied with the terms and conditions for the source of all data.

### Methodology

#### PCA

The principal Components Analysis (PCA) was first introduced to non-random variables by Pearson (1901), and then extended to random vectors [15]. Nowadays, it’s the most widely used dimension-reduction method [16].

PCA is an algorithm to transform the columns of a dataset into a new set of features called Principal Components, which contain less variables and retain as much information about the original variable as possible. Specifically, the PCA extracts diffusion indexes $F_{t}^{PCA}$ as a weighted sum of predictors X_i,t_, which can be expressed as follows:

$$X_{i,t}=\lambda_{i}^{\prime }F_{t}^{PCA}+e_{i,t}.$$ (2.11)

With the PCA diffusion indexes $F_{t}^{PCA}$, we can predict the target as:

$${\tilde{y}}_{t+h}^{PCA}={\tilde{\alpha }}^{PCA}+\sum_{k=1}^{K}({\tilde{\beta }}_{k,t}^{PCA})\prime F_{k,t}^{PCA}.$$ (2.12)

In this way, a large chunk of the information across the full dataset is effectively compressed into fewer feature columns. This allows for dimensionality reduction and the ability to visualize the separation of classes or clusters if any. However, PCA is an unsupervised learning technique that ignores the prediction target in the prediction process. Therefore, the forecasting result of PCA may not be stable. In the extreme cases, when factors are strong, PCA cannot distinguish between the target-relevant and irrelevant latent factors. When the factors are weak, PCA could fail to extract the signals from the large amount of noise, resulting in biased forecasts when all factors are used [8].

#### s-PCA

The scaled PCA (s-PCA) is a novel dimensionality reduction method proposed by Huang et al. [8], which modifies the traditional PCA by considering the prediction target. In particular, the s-PCA tends to down-weight those predictors with weak forecasting power and overweight those with strong forecasting power. As a result, the s-PCA could overcome the deficiencies of PCA to identify predictors that are particularly useful for predicting targets and obtain more significant forecast.

Specifically, the s-PCA extracts diffusion indexes in two steps. In the first step, we develop a panel of scaled predictors, (γ_1_X_i,t_,⋯,γ_N_X_N,t_), where the scaled coefficient γ_1_ denotes the estimated slope through regressing the prediction target y_i,t_ on the corresponding (standardized) indicators X_i,t_:

$$y_{t+1}=\alpha_{i}+\gamma_{i}X_{i,t}+\varepsilon_{t+1}.$$ (2.13)

In the second step, we apply PCA to the scaled predictors to extract s-PCA diffusion indexes $F_{t}^{s-PCA}$ as the new predictors:

$$\gamma_{i}X_{i,t}=\lambda_{i}^{\prime }F_{t}^{s-PCA}+e_{i,t}.$$ (2.14)

Finally, we could predict the target using the s-PCA diffusion indexes as:

$${\hat{y}}_{t+h}^{s-PCA}={\hat{\alpha }}^{s-PCA}+\sum_{k=1}^{K}({\tilde{\beta }}_{k,t}^{s-PCA})\prime F_{k,t}^{s-PCA}.$$ (2.15)

Because the target y_t+h_ depends on the predictors instead of the loadings, the s-PCA method has a large chance to outperform the PCA method, especially when all factors are used. Kelly et al. [17], Pelger [18], Gu et al. [19], Lettau and Pelger [20,21] applied similar methods and demonstrated that the s-PCA can yield satisfactory results in various areas.

#### PLS

In accordance with the s-PCA, partial least squares (PLS) is a supervised learning method that uses the prediction target to discipline its dimension reduction [22–24].

Specifically, the PLS method extracts diffusion indexes $F_{k,t}^{PLS}$ in two steps. In the first step, we regress each predictor (X_i,t_,⋯,X_N,t_) on the prediction target:

$$X_{i,t-1}=\varphi_{i,0}+\varphi_{i}y_{t}+u_{t-1}.$$ (2.16)

In the second step, we extract PLS diffusion indexes $F_{t}^{PLS}$ through running a time-series regression for each predictor (X_i,t_,⋯,X_N,t_) and the corresponding ${\hat{\varphi }}_{i}$ estimated in Eq (2.16):

$$X_{i,t}=\theta_{t}+F_{t}^{PLS}{\hat{\varphi }}_{i}+\upsilon_{t-1}.$$ (2.17)

Finally, we could predict the target using the PLS diffusion indexes estimated in Eq (2.17):

$${\hat{y}}_{t+h}^{PLS}={\hat{\alpha }}^{PLS}+\sum_{k=1}^{K}({\tilde{\beta }}_{k,t}^{PLS})\prime F_{k,t}^{PLS}.$$ (2.18)

The PLS method makes dependent variable connect tightly with the independent variable and thus may achieve satisfactory results. Kelly and Pruitt [23], Light et al. [25] found that the PLS method exhibited strong forecasting power even with relatively small data.

#### LSTM

As a special form of recurrent neural network (RNN), long-short term memory (LSTM) neural network is able to handle the long-term dependence of time-series data well. The LSTM neural network structure contains a series of recurrently connected sub-networks (i.e., memory modules), each of which contains one or more self-connecting cells, as well as a system of three gating units controlling information flow (input gate, output gate, and forget gate). Specifically, the execution steps in an LSTM network can be summarized as follows:

Firstly, determine the information that needs to be extracted from the cell through the forget gate (*f*_*t*_):

$$f_{t}=\sigma (b_{f}+W_{f}x_{t}+U_{f}h_{t-1}),$$ (2.19)

whereby σ is a sigmoid activation function that sets the information flow weight to a value between 0 and 1. 0 means that the information is completely deleted and 1 means that all information is retained. x_t_ is the current input vector and h_t_ is the current hidden layer vector. b_f_, W_f_, and U_f_ are the bias, input weights, and loop weights of the forget gate, respectively.

Next, update the state of information in the cell. Let *g*_*t*_ be an input gate between 0 and 1 controlled by the sigmoid activation function:

$$g_{t}=\sigma (b_{g}+W_{g}x_{t}+U_{g}h_{t-1}).$$ (2.20)

Then the updated cell state C_t_ on the basis of C_t−1_ is:

$$C_{t}=f_{t}^{*}C_{t-1}+g_{t}^{*}\tanh\left(b_{c}+W_{c}x_{t}+U_{c}h_{t-1}\right).$$ (2.21)

Lastly, the message output controlled by the output gate o_t_ is:

$$h_{t}=o_{t}^{*}\tanh\left(C_{t}\right),$$ (2.22)

whereby the detailed output gate controlled by sigmoid activation function is:

$$o_{t}=\sigma (b_{o}+W_{o}x_{t}+U_{o}h_{t-1}).$$ (2.23)

In conclusion, LSTM model contains not only the external loop between the hidden layer cells involved in RNN, but also the self-loop within the cells. Because of this special structure, LSTM model can reflect the nonlinearity of the financial time series data and the complex interactions between features. Therefore, LSTM model may have higher prediction accuracy compared to traditional econometric models and other machine learning algorithms.

### Empirical analysis model evaluation metrics

The coefficient of determination (R2), the root mean square error (RMSE) and the mean absolute error (MAE) are three widely used metrics to evaluate the performance of the prediction model. Among them, R2 indicates the degree of fitting to the actual value, whose value ranges between 0 and 1. Models with R2 values closer to 1 perform better. RMSE indicates the deviation between the predicted value and the true value. MAE measures the average absolute error between the predicted value and the true value. The smaller the RMSE values and MAE values are, the better the performance of the prediction model is. The three evaluation metrics are calculated as follows:

$$R^{2}=1-\frac{\sum_{t=1}^{N}{\left(y_{t}-{\hat{y}}_{t}\right)}^{2}}{\sum_{t=1}^{N}{\left(y_{t}-{\bar{y}}_{t}\right)}^{2}},$$ (3.1)

$$RMSE=\sqrt{\frac{1}{N}\sum_{t=1}^{N}{\left(y_{t}-{\hat{y}}_{t}\right)}^{2}},$$ (3.2)

$$MAE=\frac{1}{N}\sum_{t=1}^{N}\left|y_{t}-{\hat{y}}_{t}\right|,$$ (3.3)

where $y_{t},{\hat{y}}_{t},{\bar{y}}_{t}$ denote the true value, predicted value and average value at time t, respectively. N is the number of data points.

In addition, we follow Campbell and Thompson [26] and employ $R_{OS}^{2}$ to evaluate the out-of-sample performance of the prediction model further, which is defined as follows:

$$R_{OS}^{2}=1-\frac{\sum_{t=1}^{q}{\left(y_{m+t}-{\hat{y}}_{M.m+t}\right)}^{2}}{\sum_{t=1}^{q}{\left(y_{m+t}-{\hat{y}}_{B.m+t}\right)}^{2}},$$ (3.4)

where $y_{m+t},{\hat{y}}_{M.m+t},{\hat{y}}_{B.m+t}$ represent the true value, the predicted value of the prediction model, and benchmark prediction of the historical average model at time m+t, respectively. m is the total length of the training period and validation period. q is the length of the test period. A positive $R_{OS}^{2}$ statistic implies that the prediction model has better performance than the benchmark model.

## Results and discussion

This paper employs a new dimensionality reduction method named s-PCA to predict carbon prices. In this section, we predict the carbon price in three steps. Firstly, we construct a library of indicators that affect carbon prices, including technical, financial and commodities indicators. Secondly, we apply the s-PCA method to reduce the dimensionality of the indicators. Finally, we employ traditional regression method and LSTM to predict carbon prices based on the diffusion indexes. The parameters of the LSTM model are adjusted according to the R2 of the validation set. To verify the superiority of the s-PCA method in carbon price prediction, PCA and PLS are selected for comparative analysis. Then we will treat Hubei as a research subject, in which the prediction horizon and the number of diffusion indexes are set to be 1.

### In-sample results

The in-sample results of all methods of Hubei in predicting carbon prices are shown in Tables 2 and 3. Based on all results, the analysis of each method is as follows:

In the case of prediction based on linear regression method, the prediction performance of the model based on the s-PCA method is significantly better than that based on the PCA method and the PLS method. Specifically, the R2 of the s-PCA is 71.65%, which is much larger than that of the PCA and PLS, which is 20.02% and 68.11%, respectively. In addition, the RMSE and MAE of the s-PCA are 2.69 and 2.11 respectively, which are smaller than that of the PCA and PLS, which are 4.53 and 2.86 in terms of RMSE, 3.48 and 2.24 in terms of MAE, respectively.

In the case of prediction based on the LSTM method, s-PCA method still has the highest R2 and the lowest errors, which indicates that s-PCA method perform better than the PCA method and the PLS method in carbon price prediction. Specifically, the three evaluation metrics of the s-PCA method (R2=99.67%, RMSE=0.29, MAE=0.22) are much better than the PCA method (R2=97.28%, RMSE = 0.83, MAE = 0.69) and the PLS method (R2=99.55%, RMSE = 0.34, MAE = 0.27), which shows that the s-PCA method can indeed improve the prediction accuracy of carbon prices.

Compared with the linear regression method, the LSTM model could improve the prediction performance of the s-PCA method. Specifically, the prediction based on the s-PCA method and the LSTM model achieves larger R2, as along with smaller RMSE and MAE (R2 = 99.67%, RMSE = 0.29, MAE = 0.22) than the prediction based on the s-PCA method and the linear regression method (R2 = 71.65%, RMSE = 2.69, MAE = 2.11).

As a consequence, in the in-sample analysis, the s-PCA method has a stronger performance than the PCA method and the PLS method in carbon price prediction. In addition, the LSTM model could improve the performance of the s-PCA method compared to the linear regression method.

**Table 2:** In-sample results based on linear regression.

	R2	RMSE	MAE
s-PCA	71.65%	2.69	2.11
PCA	20.02%	4.53	3.48
PLS	68.11%	2.86	2.24

**Table 3:** In-sample results based on LSTM method.

	R2	RMSE	MAE
s-PCA	99.67%	0.29	0.22
PCA	97.28%	0.83	0.69
PLS	99.55%	0.34	0.27

### Out-of-sample results

Tables 4 and 5 present the out-of-sample prediction performance of Hubei carbon price by all methods. The statistical significance for RMSE and MAE is based on the Diebold and Mariano [27] test (D-M test), in which the alternative hypothesis is that the prediction accuracy of the s-PCA method is higher than that of the benchmark model. The benchmark model is based on historical average, which is a widely used out-of-sample benchmark according to Welch and Goyal [28]. The observations can be summarized as follows:

The RMSEs and MAEs for all prediction methods are significantly small at the 1% level, which indicates that all prediction methods outperform the historical average benchmark in terms of out-of-sample RMSE and MAE. In other words, these prediction methods show strong out-of-sample forecasting capability in carbon price prediction.

In the case of out-of-sample prediction based on the linear regression method, the s-PCA method yields significantly larger $R_{OS}^{2}$ as well as smaller MASE and MAE (R2=68.94%, RMSE=1.95, MAE=1.16) than the PCA method (R2=18.06%, RMSE=4.94, MAE=2.26) and the PLS method (R2=66.87%, RMSE=2.05, MAE=1.22). The results indicate that the s-PCA method is a better dimensionality reduction method when using linear regression method to predict carbon prices.

In the case of out-of-sample prediction based on the LSTM model, the s-PCA method performs much better than the PCA method and the PLS method. As can be seen from Table 5, the $R_{OS}^{2}$ of the s-PCA method is 85.12%, which is larger than the PCA method and the PLS method. In addition, the RMSE and MAE of the s-PCA method are 0.83 and 0.60, respectively, which are significantly smaller than the other two comparative methods. The results indicate that the s-PCA method outperforms other dimensionality reduction methods when predicting carbon prices with the LSTM model.

The LSTM model can improve the prediction accuracy of the s-PCA method in carbon price prediction. By comparing three evaluation metrics of the s-PCA method with linear regression method and the s-PCA method with the LSTM model, we can find that the performance of the s-PCA method with LSTM model is much more excellent than that of the s-PCA method with linear regression method. Therefore, using the LSTM model to predict carbon prices can improve the prediction performance of the s-PCA method.

**Table 4:** Out-of-sample results based on linear regression.

	$R_{OS}^{2}$	RMSE	MAE
s-PCA	68.94%	1.95***	1.16***
PCA	18.06%	4.94***	2.26***
PLS	66.87%	2.05***	1.22***

Note: *, **, *** indicate statistical significance at 10%, 5%, 1% level, respectively.

**Table 5:** Out-of-sample results based on LSTM method.

	$R_{OS}^{2}$	RMSE	MAE
s-PCA	85.12%	0.83***	0.60***
PCA	74.79%	1.40***	0.97***
PLS	81.47%	1.23***	0.78***

Note: *, **, *** indicate statistical significance at 10%, 5%, 1% level, respectively.

In sum, the results in this section shows that consistent with the in-sample results, the s-PCA method is superior to both the PCA method and the PLS method for carbon price prediction. Moreover, the LSTM model can improve the prediction performance of the s-PCA method in terms of predictability.

## Robustness test

### Alternative proxies of carbon prices

The carbon trading volumes of the Hubei Carbon Emissions Exchange, Guangzhou Carbon Emissions Exchange, and Shanghai Environmental Energy Exchange account for more than half of the total market in China, which indicate that the carbon markets in Hubei, Guangdong, and Shanghai can be a good representative of the Chinese carbon market. For this reason, we further use the carbon prices of Guangzhou and Shanghai to test the out-of-sample performance of the s-PCA method. Tables 6 and 7 report the out-of-sample results for predicting the carbon prices of Guangzhou and Shanghai. The results show that the s-PCA method continues to perform better (i.e., larger $R_{OS}^{2}$, smaller RMSE and MAE) than other comparative methods. Moreover, the LSTM model can improve the prediction accuracy of the s-PCA method. These results prove that our out-of-sample results are robust to other proxies of carbon prices.

**Table 6:** Out-of-sample results based on linear regression for Guangzhou and Shanghai.

	Guangzhou			Shanghai		
	$R_{OS}^{2}$	RMSE	MAE	$R_{OS}^{2}$	RMSE	MAE
s-PCA	33.57%	0.33***	10.67***	30.94%	4.99***	3.81***
PCA	34.23%	0.34***	8.18***	52.47%	3.67***	2.42***
PLS	79.52%	0.80***	5.92***	60.52%	2.70***	1.50***

Note: *, **, *** indicate statistical significance at 10%, 5%, 1% level, respectively.

**Table 7:** Out-of-sample results based on LSTM for Guangzhou and Shanghai.

	Guangzhou			Shanghai		
	$R_{OS}^{2}$	RMSE	MAE	$R_{OS}^{2}$	RMSE	MAE
s-PCA	67.84%	7.42***	6.01***	60.15%	2.73***	1.50***
PCA	75.09%	6.53***	3.50***	62.78%	2.64***	1.47***
PLS	89.95%	4.15***	2.55***	74.73%	1.62***	1.05***

Note: *, **, *** indicate statistical significance at 10%, 5%, 1% level, respectively.

### Different prediction horizons

Huang et al. [8] argue that it is possible to achieve satisfactory prediction results by chance after data mining on prediction horizons. To alleviate this concern, this paper further choose another four prediction horizons to test the performance of the s-PCA method. Specifically, we set the prediction horizon in [3,6,9,12].

Tables 8 and 9 report the forecasting results for different prediction horizons. It can be seen that for any of the four prediction horizons, the s-PCA method generates larger $R_{OS}^{2}$, smaller RMSE and MAE than the other comparative methods. In addition, the s-PCA method combined with the LSTM model achieve better performance than the s-PCA method combined with the linear regression model. These results all prove that the out-of-sample results are robust to different prediction horizons.

**Table 8:** Out-of-sample results based on Linear Regression for different prediction horizons.

	prediction horizon=3			prediction horizon=6		
	$R_{OS}^{2}$	RMSE	MAE	$R_{OS}^{2}$	RMSE	MAE
s-PCA	35.15%	3.95***	2.27***	24.99%	4.92***	2.24***
PCA	42.28%	3.60***	2.17***	35.27%	4.53***	2.02***
PLS	64.32%	2.67***	1.99***	53.26%	3.66***	1.98***
	prediction horizon=9			prediction horizon=12		
	$R_{OS}^{2}$	RMSE	MAE	$R_{OS}^{2}$	RMSE	MAE
s-PCA	45.66%	3.86***	2.78***	45.56%	3.87***	2.81***
PCA	49.83%	3.86***	2.74***	48.88%	3.74***	2.67***
PLS	62.92%	3.66***	2.27***	56.58%	2.97***	1.99***

Note: *, **, *** indicate statistical significance at 10%, 5%, 1% level, respectively.

**Table 9:** Out-of-sample results based on LSTM for different prediction horizons.

	prediction horizon=3			prediction horizon=6		
	$R_{OS}^{2}$	RMSE	MAE	$R_{OS}^{2}$	RMSE	MAE
s-PCA	71.43%	1.61***	1.50***	63.48%	2.45***	1.39***
PCA	84.61%	1.06***	1.13***	79.81%	1.48***	1.07***
PLS	89.73%	0.56***	0.89***	88.92%	1.04***	0.70***
	prediction horizon=9			prediction horizon=12		
	$R_{OS}^{2}$	RMSE	MAE	$R_{OS}^{2}$	RMSE	MAE
s-PCA	71.20%	3.16***	1.83***	60.96%	2.25***	1.58***
PCA	86.74%	2.10***	1.34***	86.09%	1.28***	1.08***
PLS	95.39%	1.09***	0.85***	89.06%	1.01***	0.86***

Note: *, **, *** indicate statistical significance at 10%, 5%, 1% level, respectively.

### Alternative forecasting window size

Following Sun and Huang [4], Zhou and Wang [13], we consider another forecasting window size by dividing the data set into a training set (80%), a validation set (10%), and a test set (10%).

Tables 10 and 11 report the out-of-sample results for alternative forecasting window size. We observe that all of the prediction methods always generate significant $R_{OS}^{2}s$, RMSEs and MAEs. Among these methods, the s-PCA method has stronger prediction performance (larger $R_{OS}^{2}$, smaller RMSE and MAE) than other competing methods. Furthermore, the LSTM model can improve the prediction performance of the s-PCA method by generating larger $R_{OS}^{2}$, RMSE and MAE. This is consistent with our results when dividing the data set into a training set (60%), a validation set (20%), and a test set (20%). Hence, the out-of-sample prediction results are robust to alternative forecasting window size.

**Table 10:** Out-of-sample results based on linear regression for alternative forecasting window size.

	$R_{OS}^{2}$	RMSE	MAE
s-PCA	49.09%	4.50***	4.56***
PCA	52.38%	4.14***	4.21***
PLS	60.78%	3.74***	3.51***

Note: *, **, *** indicate statistical significance at 10%, 5%, 1% level, respectively.

**Table 11:** Out-of-sample results based on LSTM for alternative forecasting window size.

	$R_{OS}^{2}$	RMSE	MAE
s-PCA	69.91%	3.49***	2.78***
PCA	84.82%	2.76***	1.98***
PLS	90.94%	2.14***	1.03***

Note: *, **, *** indicate statistical significance at 10%, 5%, 1% level, respectively.

### Different size of diffusion index

According to the empirical analysis in the previous section, we can learn that the s-PCA method is superior to other comparative methods based on the first diffusion index. In order to test the robustness of the out-of-sample prediction performance for the s-PCA method, we employ the first and second diffusion indexes to re-predict the carbon price based on all forecasting methods.

The out-of-sample prediction results are reported in Tables 12 and 13 when we use the first and second diffusion indexes. It can be seen that the s-PCA method outperforms other comparative methods with larger $R_{OS}^{2}$, smaller RMSE and MAE. Furthermore, compared with the s-PCA method with the linear regression method, the s-PCA method with the LSTM model performs better, indicating that the LSTM model can improve the prediction performance of the s-PCA method. Overall, the out-of-sample results suggest that the prediction performance of the s-PCA method is robust when we use the first and second diffusion indexes.

**Table 12:** Out-of-sample results based on linear regression for different size of diffusion index.

	$R_{OS}^{2}$	RMSE	MAE
s-PCA	21.65%	0.21***	4.71***
PCA	48.30%	0.49***	3.25***
PLS	61.90%	0.63***	2.65***

Note: *, **, *** indicate statistical significance at 10%, 5%, 1% level, respectively.

**Table 13:** Out-of-sample results based on LSTM method for different size of diffusion index.

	$R_{OS}^{2}$	RMSE	MAE
s-PCA	70.66%	0.70***	1.55***
PCA	86.46%	0.87***	1.39***
PLS	89.01%	0.90***	0.79***

Note: *, **, *** indicate statistical significance at 10%, 5%, 1% level, respectively.

## Market timing

In contrast to studying the statistical significance of carbon price prediction, it is more meaningful for investors to study its economic significance, which could give them investment advice and generate possible profits. Following He et al. [29], we further study the economic benefits of the s-PCA method from the perspective of market timing.

In this study, we will take a long position at the time t if the carbon price at time t+30 is higher than the carbon price at time t. Otherwise, we will take a short position. At the end of time t+30, we will close the position we took. The market timing strategy based on the carbon price prediction for time t can be expressed as follows:

$$A(t)=\left\{ \begin{array}{c} 1,if\;P_{t+30}>P_{t}\\ -1,otherwise \end{array}\right.,$$ (5.1)

where A(t) is the action we take at time t, 1 denotes that we take a long position, and -1 denotes that we take a short position.

To evaluate the performance of the market timing strategy, we consider the Buy-and-Hold strategy as a benchmark strategy, which takes a long position at time t, and close the position at time t+30.

Table 14 reports the market timing results for carbon prices. Here, the average return is annualized and in percentage. It can be seen that the s-PCA method has the largest average returns in all methods and market timing strategies, which is 38.14% based on the linear regression method and 70.39% based on the LSTM model, respectively. However, the risks of the s-PCA method, which are measured by standard deviation, are also much higher than most other market timing strategies, which is 12.66% based on the linear regression method and 12.26% based on the LSTM model, respectively. When we take risk into consideration, the performance of a market timing strategy can be measured in terms of the Sharpe ratio. We observe that the Sharpe ratio of the s-PCA method with the linear regression method is 3.93 and that of the s-PCA method with the LSTM model is 7.49. These Sharpe ratios are much higher than those of most other market timing strategies, except for the Sharpe ratio of the PLS method with the LSTM model, which is 7.68.

**Table 14:** Market timing results for carbon prices.

	Prediction method	Average return(%)	Standard deviation (%)	Sharpe ratio
s-PCA	Linear Regression	38.14	12.66	3.93
	LSTM	70.39	12.26	7.49
PCA	Linear Regression	4.35	6.57	0.96
	LSTM	20.81	11.91	2.28
PLS	Linear Regression	14.52	9.17	2.07
	LSTM	70.27	11.93	7.68
Buy-and-Hold		37.05	14.84	3.25

In a word, the s-PCA method is of greater economic importance compared to other market timing strategies. Moreover, the LSTM model can improve the performance of the s-PCA method in terms of the market timing strategy.

## Conclusions and future work

In this paper, we employ the s-PCA model proposed by Huang et al. [8] to predict carbon price with 71 technical indicators, 13 financial indicators and 25 commodity indicators. First, we construct a factor library in which indicators are likely to have an impact on the carbon price. Second, we use the s-PCA method to reduce the dimensionality of the influencing factors. Third, after dimensionality reduction, we employ the linear regression method and the LSTM model to predict the carbon price. Fourth, we examine the economic significance of the s-PCA method from a market timing perspective.

Using the carbon price of Hubei for empirical analysis, the prediction performance of the s-PCA method has been compared with the PCA method and the PLS method. The empirical results show that the s-PCA method is superior to other comparative methods from both a statistical perspective and an economic perspective. Specifically, the s-PCA method yields larger R^2^, smaller RMSE and MAE in both in- and out-of sample analysis. In addition, the LSTM model can provide significant improvements to the s-PCA method for carbon price prediction, which may due to its properties of long-term memory and nonlinearity. Our results are robust to a series of settings, including different carbon markets, different forecasting horizons, alternative forecasting window size, and different size of diffusion index. From the perspective of market timing, an investor can achieve higher average return and Sharpe ratio by applying the s-PCA method than applying other comparative strategies.

In the future, (a) we should look into more advanced prediction models to further improve the prediction performance of the s-PCA method. (b) We should construct more realistic investment strategies and provide more useful advice to investors. (c) We need to analyze the performance of the s-PCA method in other carbon markets.

## Supporting information

S1 File (XLS)
