# Correction: Carbon price prediction based on a scaled PCA approach

**DOI:** 10.1371/journal.pone.0329804

**Published:** 2025-08-05

**Authors:** The PLOS One Staff

## Notice of republication

An earlier, incomplete version of this article was published in error. The publisher apologizes for the error. This article was republished on June 5, 2025, to correct the error. Please download the article again to view the corrected version. Both the originally published, uncorrected article and the republished, corrected article are provided here for reference.

## Supporting information

S1 File Originally published, uncorrected article. (PDF)

S2 File Republished, corrected article. (PDF)
